# CXCR4 Recognition by L- and D-Peptides Containing the Full-Length V3 Loop of HIV-1 gp120

**DOI:** 10.3390/v15051084

**Published:** 2023-04-28

**Authors:** Ruohan Zhu, Xiaohong Sang, Jiao Zhou, Qian Meng, Lina S. M. Huang, Yan Xu, Jing An, Ziwei Huang

**Affiliations:** 1 School of Life Sciences, Tsinghua University, Beijing 100084, China; 2 Ciechanover Institute of Precision and Regenerative Medicine, School of Medicine, Chinese University of Hong Kong, Shenzhen 518172, China; 3 Division of Infectious Diseases and Global Public Heath, Department of Medicine, School of Medicine, University of California at San Diego, 9500 Gilman Drive, La Jolla, CA 92093, USA

**Keywords:** HIV-1 gp120 V3 loop, chemokine receptor CXCR4, molecular recognition, D-peptide, HIV-1 entry

## Abstract

Human immunodeficiency virus-1 (HIV-1) recognizes one of its principal coreceptors, CXC chemokine receptor 4 (CXCR4), on the host cell via the third variable loop (V3 loop) of HIV-1 envelope glycoprotein gp120 during the viral entry process. Here, the mechanism of the molecular recognition of HIV-1 gp120 V3 loop by coreceptor CXCR4 was probed by synthetic peptides containing the full-length V3 loop. The two ends of the V3 loop were covalently linked by a disulfide bond to form a cyclic peptide with better conformational integrity. In addition, to probe the effect of the changed side-chain conformations of the peptide on CXCR4 recognition, an all-D-amino acid analog of the L-V3 loop peptide was generated. Both of these cyclic L- and D-V3 loop peptides displayed comparable binding recognition to the CXCR4 receptor, but not to another chemokine receptor, CCR5, suggesting their selective interactions with CXCR4. Molecular modeling studies revealed the important roles played by many negative-charged Asp and Glu residues on CXCR4 that probably engaged in favorable electrostatic interactions with the positive-charged Arg residues present in these peptides. These results support the notion that the HIV-1 gp120 V3 loop-CXCR4 interface is flexible for ligands of different chiralities, which might be relevant in terms of the ability of the virus to retain coreceptor recognition despite the mutations at the V3 loop.

## 1. Introduction

CXC chemokine receptor 4 (CXCR4) belongs to class A G-protein-coupled receptors (GPCRs) [1]. It has a unique cognate ligand CXC chemokine ligand 12 (CXCL12), also called stromal-cell-derived factor (SDF-1α) [2,3]. The CXCR4/CXCL12 axis plays important roles in complex biological processes such as inflammatory response, the mobilization and homing of hematopoietic stem cells (HSPCs), angiogenesis, and cell survival [4]. CXCR4 is also shown to be a cell surface receptor of ubiquitin and macrophage migration inhibitory factor (MIF) and can be partially activated upon interaction [5,6,7].

In 1996, CXCR4 was identified, along with another chemokine receptor CCR5, as the long sought after co-receptor for human immunodeficiency virus type 1 (HIV-1) [2,8,9,10]. It is thought that the envelope glycoprotein 120 (gp120) of HIV-1 binds the host cell’s primary receptor CD4 and undergoes conformation changes which expose its third variable loop (V3 loop) to interact with coreceptor CXCR4 or CCR5, triggering further molecular events that lead to virus–cell membrane fusion and infection [11,12]. The co-receptor usage depends on the cell tropism of an HIV-1 strain, with CXCR4 as the coreceptor for T-cell-line-tropic (T-tropic) HIV-1 and CCR5 for macrophage-tropic (M-tropic) HIV-1 [13]. Usually, viruses using CCR5 as the coreceptor (i.e., R5 viruses) are predominant during the early stage of the infection, whereas viruses using CXCR4 (X4 viruses) are often associated with a late and rapid clinical progression [14,15]. The diverse functions of CXCR4 in physiologies and pathologies as described above have made it a therapeutic target in intensive drug discovery research for HIV and other human diseases [16].

For more than two decades, our laboratories have been interested in the stereochemistry of CXCR4-ligand recognition and its implication in the molecular mechanism of HIV-1 entry via this coreceptor. In 2002, we reported the original finding that CXCR4 could recognize both L- and D-peptides of identical amino acid sequences but with opposite chirality, corresponding to the N-terminus of a viral chemokine vMIP-II, which revealed the flexible stereochemical requirement on the CXCR4-chemokine ligand interface [17]. More recently, we extended this investigation to the viral ligand of CXCR4, HIV-1 gp120, and found that L- and D-peptides derived from fragments of HIV-1 gp120 V3 loop, when combined with another D-peptide derived from vMIP-II to gain higher receptor binding affinity, displayed nanomolar affinity for CXCR4 and strong antagonistic activities [18,19], which suggested that the flexible stereochemistry initially observed for the CXCR4-chemokine peptide interface appeared to hold true for the interaction between CXCR4 and HIV-1 gp120 V3 loop peptide fragments.

Here, in this study, we wanted to further extend the above-described observation with fragments of HIV-1 gp120 V3 loop to the full-length V3 loop. Two L- and D-peptides corresponding to the full-length gp120 V3 loop of HIV-1 89.6 (dual-tropic) strain were chemically synthesized and tested for their interaction with CXCR4 using an anti-CXCR4 antibody competitive binding assay. Both peptides bound CXCR4 with comparable IC_50_ values. The molecular mechanisms of CXCR4’s interactions with these two peptides were investigated by molecular dynamics (MD) simulations. These results and their implications for understanding the mechanisms of the entry of HIV-1 via CXCR4 and its evasion of antibody detection are discussed.

## 2. Results and Discussion

### 2.1. Design of Cyclic L- and D-Peptides Corresponding to the Full-Length V3 Loop of gp120 of the Dual-Tropic HIV 89.6 Strain

**Figure 1:**
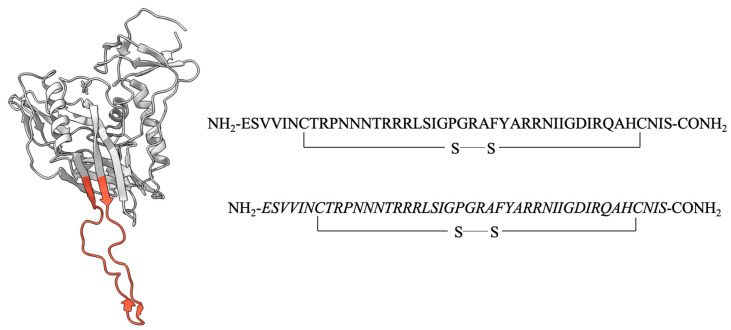
A pair of L- and D-peptides corresponding to the full-length V3 loop of HIV-1 gp120. The V3 loop of the crystal structure (PDB: 2B4C) of HIV-1 gp120 is highlighted in red. The amino acid sequence of the two L- and D-peptides which correspond to the full-length V3 loop of gp120 of the HIV-1 89.6 strain is shown. These two peptides have the same sequence, as shown with the D-peptide of all D-amino acids. These peptides are cyclized through a disulfide bond between the two cysteines as shown.

**Figure 2:**
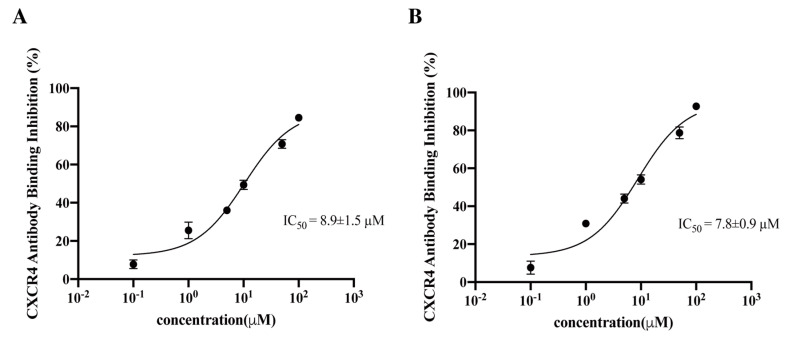
The CXCR4 competitive binding activity of cyclic (**A**) L- and (**B**) D-peptides containing the entire V3 loop sequence of gp120 of the dual-tropic HIV 89.6 strain. The IC_50_ values were determined by the competitive binding assay using an anti-CXCR4 mAb 12G5. The results were from three independent experiments and presented as means ± standard deviation.

**Figure 3:**
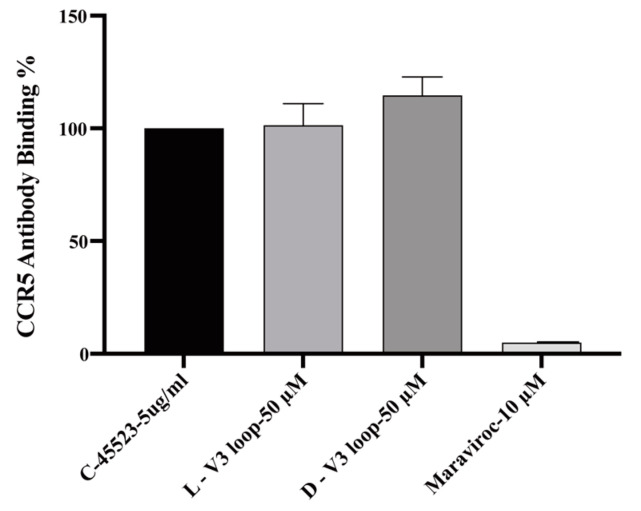
Lack of CCR5 competitive binding of the cyclic L- and D-V3 loop peptides. Maraviroc, a known CCR5 binding drug, was used as the positive control. The results were from three independent experiments.

Two cyclic L- and D-peptides containing the amino acid sequence of the entire V3 loop of gp120 of the HIV-1 89.6 strain were chemically synthesized and examined for CXCR4 competitive binding activity (Figure 1). In these peptides, a disulfide bond commonly used for peptide cyclization and conformational restraint was introduced between the two Cys residues with the goal of stabilizing the V3 loop conformation critical for receptor binding. Both peptides displayed competitive binding to CXCR4 with the IC_50_ values of 8.95 μM and 7.8 μM, respectively (Figure 2). The CCR5 competitive binding assay was also performed on these cyclic L- and D-V3 loop peptides. Neither peptides displayed any competitive binding to CCR5 (Figure 3), suggesting that their CXCR4 binding was selective.

### 2.2. Molecular Modeling of CXCR4 Interactions with Cyclic L- and D-V3 Loop Peptides

**Figure 4:**
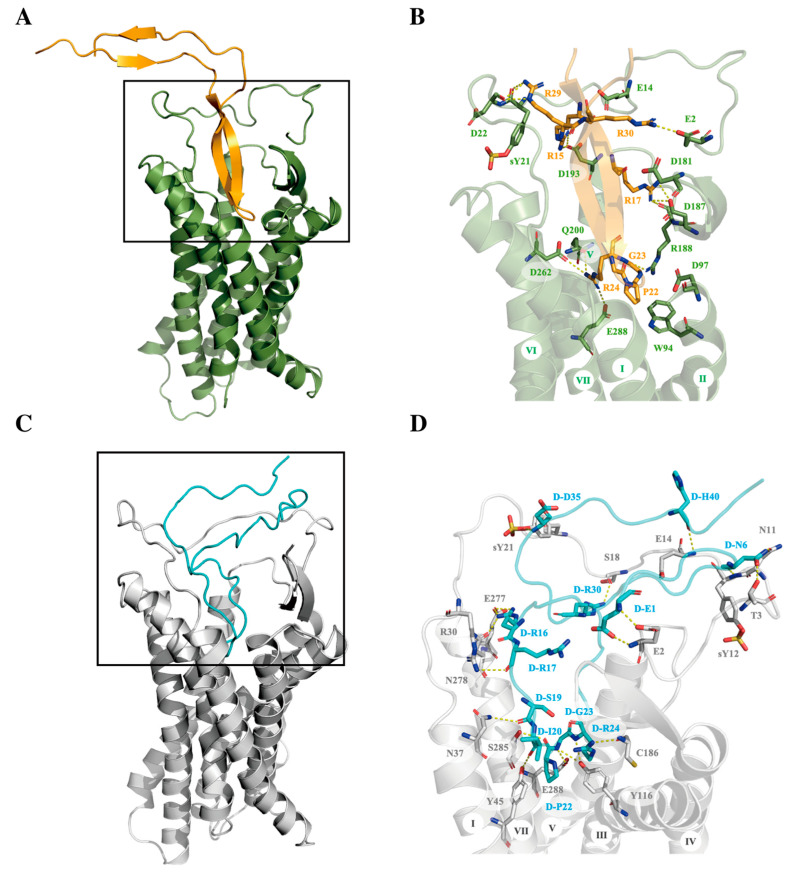
The predicted binding modes of cyclic L-V3 (**A**,**B**) and D-V3 (**C**,**D**) loop peptides in CXCR4 crystal structure (PDB: 4RWS). (**A**,**C**) Cyclic L- and D-V3 loop peptides are shown in orange and cyan cartoon models, respectively. (**B**,**D**) Key residues in interaction are shown as sticks.

To understand the structural basis of CXCR4 recognition by these peptides of opposite chirality, molecular modeling of the docking interactions of the peptides with CXCR4 was conducted using the ROSETTA FlexPepDock procedure. The resulting docking models were further refined with a 100 ns molecular dynamics (MD) simulation. The final models revealed that both L- and D-V3 peptides formed a β-hairpin that inserted into CXCR4 and made contact with nearly all of the seven transmembrane helices (Figure 4). The fragments of the peptides corresponding to the stem and base regions of the native V3 loop interacted with the extracellular loops and N-terminus of CXCR4. On the other hand, there were noticeable differences in the CXCR4 docking modes between L- and D-V3 peptides. The L-V3 peptide maintained the anti-parallel β-strand conformation during docking into the receptor, whereas in the D-V3 peptide this anti-parallel β-strand conformation became distorted, probably due to the changed chirality and, consequently, the changed side-chain orientation. In addition, the peptide fragments corresponding to the stem and base regions of the native V3 loop extended to helices V and VI for the L-V3 peptide, and to helices IV and V for the D-V3 peptide.

**Figure 5:**
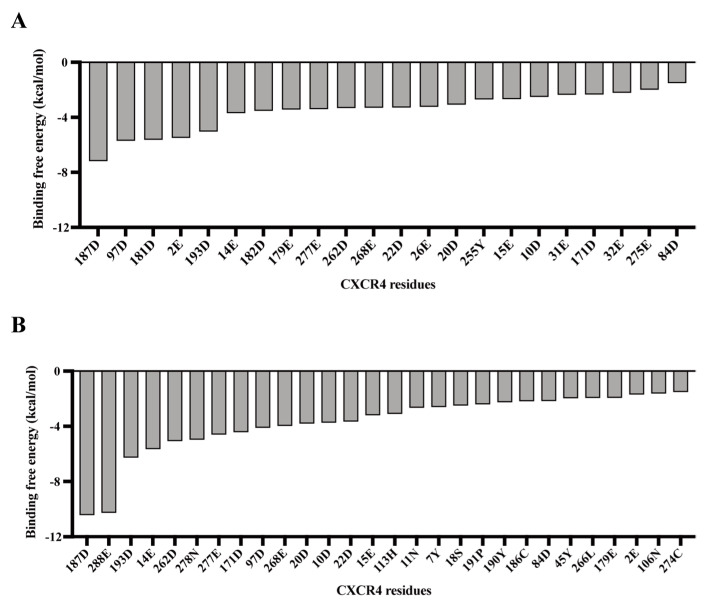
The contribution to ligand–receptor binding free energy of CXCR4 residues for the cyclic L-V3 peptide (**A**) and D-V3 peptide (**B**).

To investigate further the binding recognition mechanism of L- and D-V3 peptides with CXCR4, we employed the PDLD/S-LRA/β method to analyze the contribution of CXCR4 residue to the binding free energy of the peptide ligands (Figure 5). Many negative-charged residues, Asp and Glu, on CXCR4 were seen to contribute to the binding free energy of the receptor and the L- and D-V3 peptides, which seemed to be consistent with the roles of these negative-charged residues in electrostatic interactions with the many positive-charged Arg residues in both peptides. On the other hand, the rank orders of the residue’s contribution to the binding free energy differed between these two peptides, which was consistent with the observed differences in their receptor binding modes as described above.

## 3. Conclusions

In this study, we investigated the stereochemical mechanism of the molecular recognition of HIV-1 by coreceptor CXCR4 by using synthetic peptide probes containing the entire V3 loop of gp120, which is the major site of the virus–coreceptor interaction. To help retain the conformational integrity of the peptide, a disulfide bond that formed between the two ends of the V3 loop was used to cyclize the peptide. In addition, to probe the effect of changed side-chain conformations of the peptide on CXCR4 recognition, an all-D-amino acid analog of the L-V3 loop peptide was generated. Both of these cyclic L- and D-V3 loop peptides displayed comparable binding recognition to the CXCR4 receptor, but not another chemokine receptor, CCR5, suggesting their CXCR4-selective interactions. Further molecular modeling studies revealed the important roles played by many negative-charged Asp and Glu residues on CXCR4 that probably engaged in favorable electrostatic interactions with the positive-charged Arg residues present in these peptides. These results are in line with our recently published data on other V3 loop fragment peptides [19]. Together, they provide further support to the notion that the seemingly flexible V3 loop-CXCR4 interface might be implicated in the viral entry and immune evasion mechanisms. Specifically, with its well-known highly mutable gp120 V3 loop, HIV-1 may evade antibody recognition, which is conformation-dependent, through conformational changes caused by residual mutations reminiscent of the conformational changes caused by residual chirality changes (i.e., L- to D-amino acid residue conversion). At the same time, because of the flexible CXCR4 ligand binding surface able to accommodate residual chirality or conformational changes, HIV-1 can still retain its ability to recognize an important host cell receptor such as CXCR4 for entry and infection. Such insights may have practical implications on the rational design of effective antiviral agents. For example, peptides containing D-amino acids are generally known to be more enzymatically stable than those of L-amino acids. The accommodation of D-amino acids by the CXCR4 ligand binding surface revealed in this study provides a basis for the development of additional D-peptide analogs of high stability for antiviral application.

### 3.1. Experimental Procedures

#### 3.1.1. Peptide Synthesis

The peptides were synthesized through an Fmoc [N-(9-fluorenyl) methoxycarbonyl] chemistry solid-phase peptide synthesis approach. Fmoc-Rink Amide AM Resin (loading: 0.272 mmol/g, GL) was used as solid support. Before starting synthesis, resins were swelled in DCM (dichloromethane) for 20 min. Nα-Fmoc protecting group was removed with a DMF (N, N-Dimethylformamide) solution of 20% piperidine. 4eq Fmoc-amino acid materials were used in the coupling reaction for 60 min per single reaction. The coupling reagent was 3.8 eq HCTU (O-(6-Chloro-1-hydrocibenzotriazol-1-yl)-1,1,3,3-tetramethyluronium hexafluorophosphate) and 8eq DIEA (diisopropylethylamine) dissolved in DMF. When peptide elongating finished, the peptide was cleavage from resins with cleavage mixture which consisted of TFA (trifluoroacetic acid), phenol, H_2_O, and TIS (Triisopropylsilane) (88:2:5:5, *v*/*w*/*v*/*v*). Crude peptides were precipitated from lysate with ice-cold diethyl ether, centrifuged, and lyophilized. The crude peptides were purified by HPLC (high-performance liquid chromatography) using a preparative C18 column (water, 5 μm, 10 × 150 mm) and a linear gradient elution with acetonitrile containing 0.25% TFA. Production peaks were detected at 220 nm and 254 nm UV. Cyclization of peptides was performed by gentle stirring at 4 °C and oxidizing in air overnight; analytical HPLC (C18 column, Shimadzu, 5 μm, 4.6 × 150 mm) monitored the process of the reaction with 220 nm and 254 nm UV. The cyclic peptides with an intramolecular disulfide bond were desalted and purified by preparative HPLC again as mentioned above. The fractions containing target product were collected and lyophilized. Finally, analytical HPLC and high-resolution ESI-MS with positive mode were used to characterize purity and molecular weight, respectively, these results were shown in Supplementary Materials (Figures S1–S4).

#### 3.1.2. Competitive Binding Assay

To evaluate the co-receptor binding affinity of the peptides of interest, competitive binding assay was performed as we described before [18,20,21]. Stable CXCR4-CHO and CCR5 cell lines were constructed and cultured in DMEM medium (10% FBS, 100IU penicillin, 0.1 mg/mL streptomycin, and 200 μg/mL geneticin). Cells were collected and seeded into a 96-well plate with 5 × 10^5^ cells/well with 100μL FACS buffer (PBS with 0.5% BSA and 0.05% NaN_3_). The monoclonal antibodies used in competitive binding assay were 12G5 (final concentration 250 ng/mL, Sigma-Aldrich, MO, USA) for CXCR4 and C-45523 for CCR5 (final concentration 4 μg/mL, R&D systems, USA). The test compounds with various concentrations were added into each well and then antibody was added. After incubation for 40 min at 4 °C, cells were washed with FACS buffer and centrifugated. The secondary IgG-FITC (goat anti-mouse IgG, Sigma-Aldrich, MO, USA) antibody was added into each well and incubated for 30 min at 4 °C, then washed twice with FACS buffer by centrifugation. Fluorescence intensity (485_EX_/535_EM_) was measured by using spectrophotometric microplate reader (PerkinElmer EnVision^TM^, Waltham, MA, USA). The experimental data were collected from at least three independent experiments and analyzed by Graphpad Prism 8 and presented as means ± standard deviation.

#### 3.1.3. Peptide-CXCR4 Docking

The template structure of CXCR4 was obtained from the crystal structure (PDB:4RWS), removing chain B and mutating ^187^Cys to Asp by PyMOL. Missing residues of CXCR4 crystal structure were modeled with ChimeraX [22,23,24]. Structures of V3 were built in the web server of SWISS-MODEL [25,26,27]. D-V3 was inverted from the V3 we built before with PyMOL and then relaxed with ROSETTA. The initial complex structures for docking input were modelled based gp120-CD4-CCR5 cryo-EM structure with PyMOL [28]. The initial complex structures were relaxed by ROSETTA3 Relax protocol, and the lowest score out of the outputs was used in next step [29,30]. In order to remove internal clashes that were outside of the docking interface, the input PDB file was prepacked with side chains of each monomer before refinement with the prepack mode of FlexPepDock protocol [31]. Next, the prepacked complex structure was used as input to perform the refinement mode of FlexPepDock with 3000 independent FlexPepDock simulations (producing 3000 output PDB files). To effectively sample the conformational space, a preliminary round of centroid mode optimization was performed before refinement. Finally, the 30 lowest score (reweighted term) outputs were selected and analyzed by clustering [32].

#### 3.1.4. MD Simulation

The complex structures of V3-CXCR4 and D-V3-CXCR4 that were generated from peptide–protein docking were then employed in MD simulation. The bilayer membrane systems were built by CHARMM-GUI input generator [33]. The peptide–protein complexes were embedded in POPC lipid bilayer. The CHARMM36m force field parameters were used in all MD runs. The size of the rectangular water box was adjusted to the structure and filled with 0.15 M NaCl to neutralize the charges of systems. The CHARMM-GUI outputs files were used with default parameters for minimization and pre-equilibrium with Gromacs [34]. MD simulations of each system were performed using a constant number, pressure, and temperature (NPT) ensemble at 310 K for 100 ns in 2 fs intervals. The coordinate files of these two complexes after 100 ns MD simulations were provided in Supplementary Materials. 

#### 3.1.5. Binding Free Energy Calculation

MOLARIS-XG 9.15 software was used to calculate the binding free energy. The peptide–protein complex used in the binding free energy calculation was selected from the last frame of MD simulations described above. The binding free energy was calculated using semi-macroscopic protein dipoles Langevin dipoles-linear response approximation/β (PDLD/S-LRA/β) [35]. The relaxation was performed using the standard MOLARIS surface constrained all-atom solvent (SCAAS) boundary conditions and the local reaction field (LRF) long-range treatment. The prot_prot_bind module in MOLARIS-XG 9.15 was used to conduct the analysis. The peptide of the complex was regarded as group_a, and the residues within 60 Å of the peptides were taken as group_b.

## Data Availability

Data is contained within the article.

## Supplementary Materials

The following supporting information can be downloaded at: https://www.mdpi.com/article/10.3390/v15051084/s1, Figure S1: RP-HPLC analyzed the purity of synthesized L-V3 peptide, Figure S2: ESI-MS determined the molecular weight of synthesized of L-V3 peptide, Figure S3: RP-HPLC analyzed the purity of synthesized D-V3 peptide, Figure S4: ESI-MS determined the molecular weight of synthesized of D-V3 peptide. File S1: PDB file of L-V3 peptide bound with CXCR4 after 100ns MD simulation; File S2: PDB file of D-V3 peptide bound with CXCR4 after 100ns MD simulation.

## References

[1] Choi W.T., Yang Y.L., Xu Y., An J. (2014). Targeting Chemokine Receptor CXCR4 for Treatment of HIV-1 Infection, Tumor Progression, and Metastasis. Curr. Top. Med. Chem..

[2] Bleul C.C., Farzan M., Choe H., Parolin C., ClarkLewis I., Sodroski J., Springer T.A. (1996). The lymphocyte chemoattractant SDF-1 is a ligand for LESTR/fusin and blocks HIV-1 entry. Nature.

[3] Oberlin E., Amara A., Bachelerie F., Bessia C., Virelizier J.L., Arenzana-Seisdedos F., Schwartz O., Heard J.M., Clark-Lewis I., Legler D.F. (1996). The CXC chemokine SDF-1 is the ligand for LESTR/fusin and prevents infection by T-cell-line-adapted HIV-1. Nature.

[4] Mazo I.B., Massberg S., von Andrian U.H. (2011). Hematopoietic stem and progenitor cell trafficking. Trends Immunol..

[5] Bernhagen J., Krohn R., Lue H., Gregory J.L., Zernecke A., Koenen R.R., Dewor M., Georgiev I., Schober A., Leng L. (2007). MIF is a noncognate ligand of CXC chemokine receptors in inflammatory and atherogenic cell recruitment. Nat. Med..

[6] Saini V., Marchese A., Majetschak M. (2010). CXC chemokine receptor 4 is a cell surface receptor for extracellular ubiquitin. J. Biol. Chem..

[7] Saini V., Marchese A., Tang W.J., Majetschak M. (2011). Structural determinants of ubiquitin-CXC chemokine receptor 4 interaction. J. Biol. Chem..

[8] Feng Y., Broder C.C., Kennedy P.E., Berger E.A. (1996). HIV-1 entry cofactor: Functional cDNA cloning of a seven-transmembrane, G protein-coupled receptor. Science.

[9] Alkhatib G., Combadiere C., Broder C.C., Feng Y., Kennedy P.E., Murphy P.M., Berger E.A. (1996). CC CKR5: A RANTES, MIP-1alpha, MIP-1beta receptor as a fusion cofactor for macrophage-tropic HIV-1. Science.

[10] Trkola A., Dragic T., Arthos J., Binley J.M., Olson W.C., Allaway G.P., Cheng-Mayer C., Robinson J., Maddon P.J., Moore J.P. (1996). CD4-dependent, antibody-sensitive interactions between HIV-1 and its co-receptor CCR-5. Nature.

[11] Maddon P.J., Dalgleish A.G., McDougal J.S., Clapham P.R., Weiss R.A., Axel R. (1986). The T4 gene encodes the AIDS virus receptor and is expressed in the immune system and the brain. Cell.

[12] Wilen C.B., Tilton J.C., Doms R.W. (2012). HIV: Cell binding and entry. Cold Spring Harb. Perspect. Med..

[13] Berger E.A., Doms R.W., Fenyo E.M., Korber B.T., Littman D.R., Moore J.P., Sattentau Q.J., Schuitemaker H., Sodroski J., Weiss R.A. (1998). A new classification for HIV-1. Nature.

[14] Iwamoto A., Hosoya N., Kawana-Tachikawa A. (2010). HIV-1 tropism. Protein Cell.

[15] Connor R.I., Sheridan K.E., Ceradini D., Choe S., Landau N.R. (1997). Change in coreceptor use correlates with disease progression in HIV-1--infected individuals. J. Exp. Med..

[16] Choi W.T., Duggineni S., Xu Y., Huang Z., An J. (2012). Drug discovery research targeting the CXC chemokine receptor 4 (CXCR4). J. Med. Chem..

[17] Zhou N.M., Luo Z.W., Luo J.S., Fan X.J., Cayabyab M., Hiraoka M., Liu D.X., Han X.B., Pesavento J., Dong C.Z. (2002). Exploring the stereochemistry of CXCR4-peptide recognition and inhibiting HIV-1 entry with D-peptides derived from chemokines. J. Biol. Chem..

[18] Zhang C., Huang L.S., Zhu R., Meng Q., Zhu S., Xu Y., Zhang H., Fang X., Zhang X., Zhou J. (2019). High affinity CXCR4 inhibitors generated by linking low affinity peptides. Eur. J. Med. Chem..

[19] Zhu R., Meng Q., Zhang H., Zhang G., Huang L.S.M., Xu Y., Schooley R.T., An J., Huang Z. (2022). HIV-1 gp120-CXCR4 recognition probed with synthetic nanomolar affinity D-peptides containing fragments of gp120 V3 loop. Eur. J. Med. Chem..

[20] Xu Y., Duggineni S., Espitia S., Richman D.D., An J., Huang Z. (2013). A synthetic bivalent ligand of CXCR4 inhibits HIV infection. Biochem. Biophys. Res. Commun..

[21] Yang Y., Gao M., Zhang Q., Zhang C., Yang X., Huang Z., An J. (2016). Design, synthesis, and biological characterization of novel PEG-linked dimeric modulators for CXCR4. Bioorg. Med. Chem..

[22] Pettersen E.F., Goddard T.D., Huang C.C., Meng E.C., Couch G.S., Croll T.I., Morris J.H., Ferrin T.E. (2021). UCSF ChimeraX: Structure visualization for researchers, educators, and developers. Protein Sci..

[23] Goddard T.D., Huang C.C., Meng E.C., Pettersen E.F., Couch G.S., Morris J.H., Ferrin T.E. (2018). UCSF ChimeraX: Meeting modern challenges in visualization and analysis. Protein Sci..

[24] Webb B., Sali A. (2016). Comparative Protein Structure Modeling Using MODELLER. Curr. Protoc. Bioinform..

[25] Waterhouse A., Bertoni M., Bienert S., Studer G., Tauriello G., Gumienny R., Heer F.T., de Beer T.A.P., Rempfer C., Bordoli L. (2018). SWISS-MODEL: Homology modelling of protein structures and complexes. Nucleic Acids Res..

[26] Bienert S., Waterhouse A., de Beer T.A., Tauriello G., Studer G., Bordoli L., Schwede T. (2017). The SWISS-MODEL Repository-new features and functionality. Nucleic Acids Res..

[27] Guex N., Peitsch M.C., Schwede T. (2009). Automated comparative protein structure modeling with SWISS-MODEL and Swiss-PdbViewer: A historical perspective. Electrophoresis.

[28] Shaik M.M., Peng H., Lu J., Rits-Volloch S., Xu C., Liao M., Chen B. (2019). Structural basis of coreceptor recognition by HIV-1 envelope spike. Nature.

[29] Das R., Baker D. (2008). Macromolecular Modeling with Rosetta. Annu. Rev. Biochem..

[30] Conway P., Tyka M.D., DiMaio F., Konerding D.E., Baker D. (2014). Relaxation of backbone bond geometry improves protein energy landscape modeling. Protein Sci..

[31] Raveh B., London N., Schueler-Furman O. (2010). Sub-angstrom modeling of complexes between flexible peptides and globular proteins. Proteins.

[32] Li S.C., Ng Y.K. (2010). Calibur: A tool for clustering large numbers of protein decoys. BMC Bioinform..

[33] Jo S., Kim T., Iyer V.G., Im W. (2008). CHARMM-GUI: A web-based graphical user interface for CHARMM. J. Comput. Chem..

[34] Van Der Spoel D., Lindahl E., Hess B., Groenhof G., Mark A.E., Berendsen H.J. (2005). GROMACS: Fast, flexible, and free. J. Comput. Chem..

[35] Singh N., Warshel A. (2010). Absolute binding free energy calculations: On the accuracy of computational scoring of protein-ligand interactions. Proteins.

