# Supplementary material for: CXCR4 Recognition by L- and D-Peptides Containing the Full-Length V3 Loop of HIV-1 gp120
Source: Viruses. 2023 Apr 28;15(5):1084. doi: 10.3390/v15051084 (PMC10221217; doi:10.3390/v15051084)
Supplement: Supplementary file 1 [file viruses-15-01084-s001.zip › viruses-2345568-Supplementary materials.pdf]

Results of Peptides synthesis

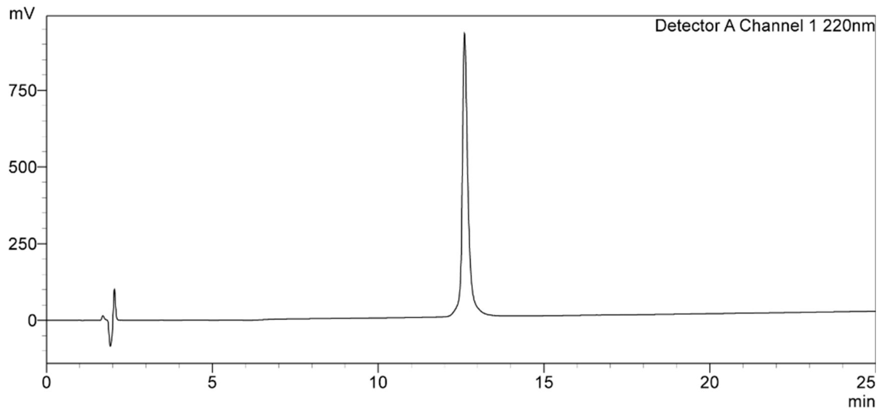

Figure S1. RP-HPLC analyzed the purity of synthesized L-V3 peptide.

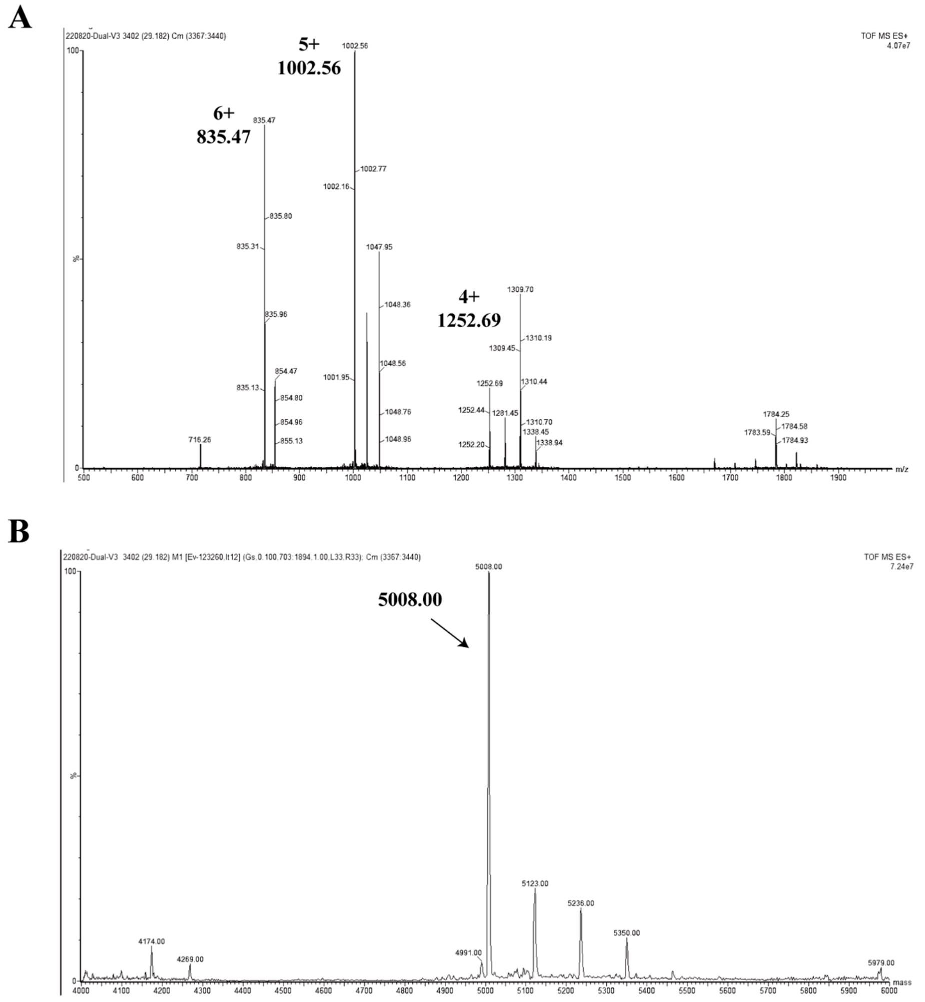

Figure S2. ESI-MS determined the molecular weight of synthesized L-V3 peptide. (A) Positive mode ESI-MS for L-V3 peptide, (B) deconvoluted ESI-MS spectra of L-V3 peptide.

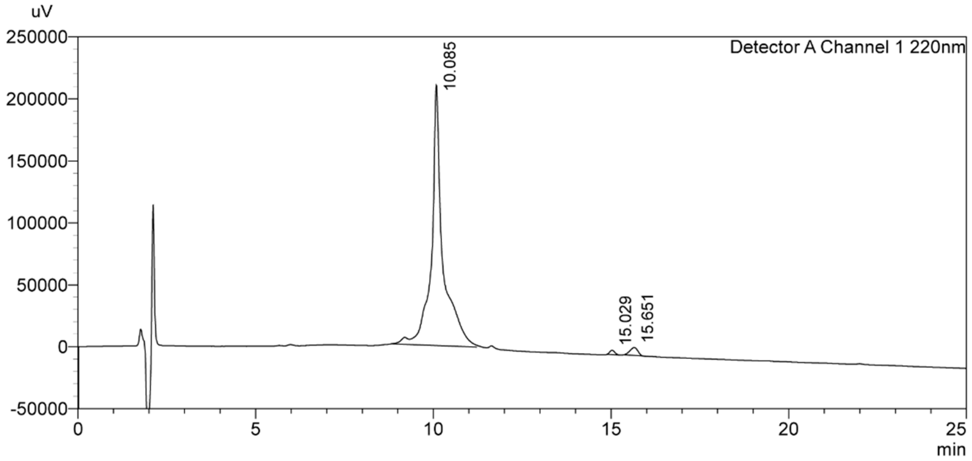

Peak#	Ret. Time	Area	Height	Conc.	Unit	Area%
1	10.085	4380271	207898	97.117		97.117
2	15.029	33479	3409	0.742		0.742
3	15.651	96561	6316	2.141		2.141
Total		4510310	217623			100.000

Figure S3. RP-HPLC analyzed the purity of synthesized D-V3 peptide.

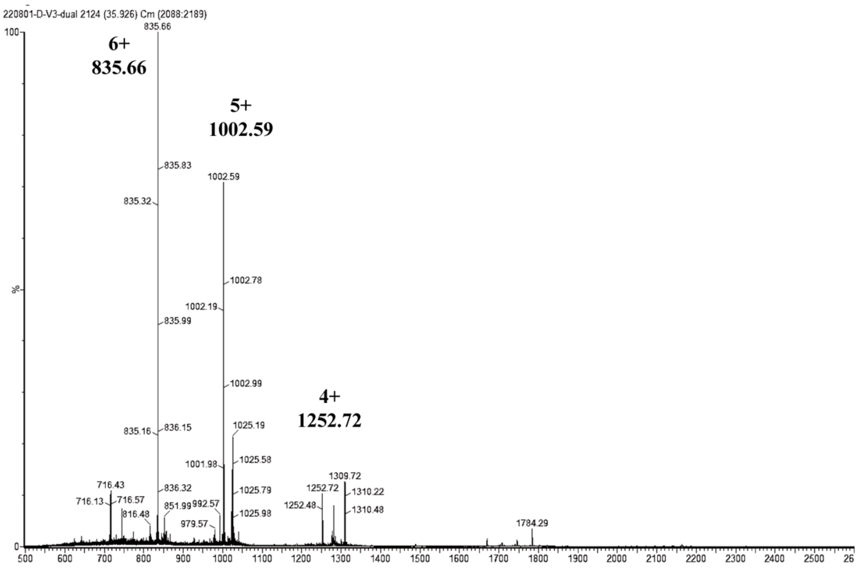

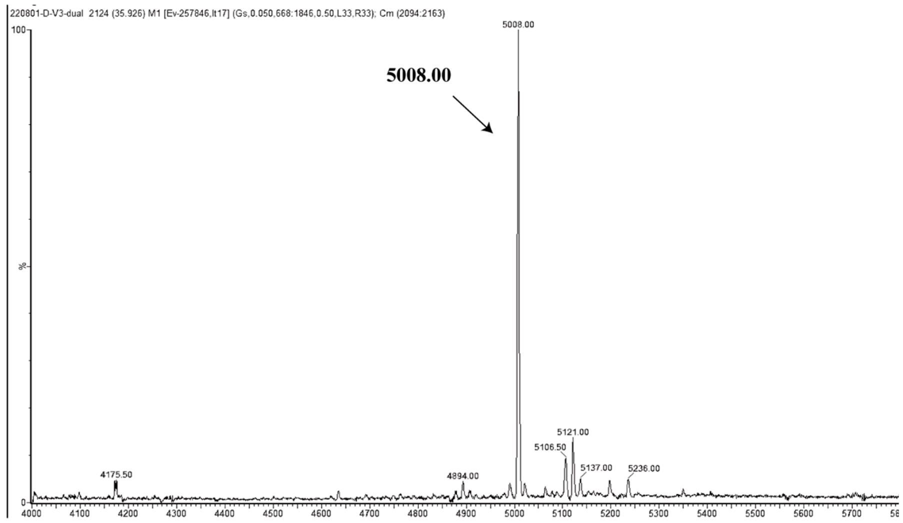

Figure S4. ESI-MS determined the molecular weight of synthesized D-V3 peptide. (A) Positive mode ESI-MS for D-V3 peptide, (B) deconvoluted ESI-MS spectra of D-V3 peptide.
